# Bastian on the Germ Theory of Disease

**Published:** 1875-06-01

**Authors:** 


### BASTIAN ON THE GERM THEORY OF DISEASE.

*From London Lancet, April,* 1875.

AT the meeting of the Pathological Society of London, on April 6th, Dr. Bastian opened the discussion by an address on the germ theory of disease, viz.: the causal relation between certain virulent inflammations or specific diseases, and the presence of low organisms in the affected tissues. He alluded to the two main theories in vogue; the one which ascribes the origin of such diseases to certain ferments, or substances allied to the ferments, which Pasteur had at one time believed and stated to be the cause of all fermentative and putrefactive processes; the other the physico-chemical theory which Liebig had advanced more than twenty years ago, and which, as it may be stated at the present day, affirms that living organisms, though they may act as ferments, act in this capacity merely by virtue of the chemical changes which the carrying on of their growth necessitates; and that other chemical changes taking place during the decay of organic matter, may make fragments of it (in the dead state) almost equally capable of initiating fermentative changes in suitable media; while in either case bacteria or allied organisms are prone to be engendered as correlative products.

In regard to the special question whether gonorrhœa, purulent ophthalmia, erysipelas, hospital gangrene, puerperal fever, pyæmia, septicæmia, and the like, are due to the presence of low organisms, he states the following facts in opposition to such a view: 1. The experiments of many investigators prove that the alleged causes of disease may be actually introduced into the blood-vessels of low animals by thousands without producing any deleterious effects in a large majority of the cases. 2. Bacteria, if not actually to be found within the blood-vessels of healthy persons, do nevertheless habitually exist in so many parts of the body in every human being, and in so many of the lower animals as to make it almost inconceivable that these organisms can be causes of disease. In healthy persons they may be found in myriads in and about the epithelium of the whole alimentary track from mouth to anus; they exist throughout the air-passages, and may be found in mucus coming from the nasal cavities, as well as in that from the minute bronchi; they are absorbed in immense numbers with the food; and lastly, in persons with open wounds, bacteria are constantly to be found in contact with such surfaces, especially if the wounds are not well cared for, though the injured person does not necessarily suffer at all in general health. 3. The argument that the bacteria of such diseases are different from those of health has yet to be proved, while, on the other hand, it has been shown that the forms which these two organisms exhibit are probably due to the peculiar conditions in which they find themselves. 4. The virulence of certain contagious mixtures diminishes in direct proportion to the increase of the bacteria in them, and again, fresh and contagious menstrua lose hardly any of their virulence by being subjected for a few minutes to a temperature of 212° F., and after being boiled in alcohol, both of which experiments, the author states, are known to be destructive to all recognized forms of living matter.

The presence of bacteria in the fluids and tissues of diseased persons, Dr. Bastian explains by the fact that certain changes have previously taken place in the constitution and vitality of such fluids and tissues, and that bacteria and allied organisms have appeared therein as pathological products.

As to the question whether the germ theory is applicable to artificial tuberculosis, syphilis, typhoid, typhus, relapsing fever, cholera, measles, scarlet fever, small-pox, and other contagious fevers, the following statements are made: 1. With two exceptions, no definite germs or organisms are to be met with in the blood of patients suffering from these diseases during any stage of their progress. 2. The virus or contagion of some of these diseases, whatever it may be, does not exhibit the properties of living matter. 3. The virus of most of these contagious diseases with which definite experiment has been made, is most potent in the fresh state, while its power diminishes in intensity as organisms reveal themselves more abundantly in it. Reference is made to the facts mentioned by Obermeier, that the blood of relapsing fever patients exhibited spirilla, and to the statements of Klein, who claims to have discovered certain peculiar bodies among the lesions of typhoid fever, especially in Peyer's patches, while there are two diseases, sheep-pox, or ovine small-pox and splenic fever, a disease occasionally communicated to persons from cattle, where it is now said there are certain low organisms in the tissues that appear to have some causal relation with the diseases. Our present notion of zymotic or ferment disease must, however, according to Dr. B., be subjected to modification, if we regard such diseases as due to the operation of low organisms in the sense that Pasteur claimed fermentation was due to the multiplication of such agents. The real fact is, that Pasteur and his followers have modified these views so considerably that it is now acknowledged that such organisms are not essential factors in fermentation, and, indeed, such processes may go on quite independently of them; fermentative changes are produced under the influence of altered chemical processes taking place in unhealthy vegetal tissue.

Some such chemical action, it is suggested, may be at work in these diseased processes, and some of the complex compounds may be thrown off, and act as contagious principles.

